# Short Linear Motifs recognized by SH2, SH3 and Ser/Thr Kinase domains are conserved in disordered protein regions

**DOI:** 10.1186/1471-2164-9-S2-S26

**Published:** 2008-09-16

**Authors:** Siyuan Ren, Vladimir N Uversky, Zhengjun Chen, A Keith Dunker, Zoran Obradovic

**Affiliations:** 1 Center for Information Science and Technology, Temple University, Philadelphia, PA 19122, USA; 2 Center for Computational Biology and Bioinformatics, Department of Biochemistry and Molecular Biology, Indiana University School of Medicine, Indianapolis, IN 46202, USA; 3 Institute for Intrinsically Disordered Protein Research, Indiana University School of Medicine, Indianapolis, IN 46202, USA; 4 Institute for Biological Instrumentation, Russian Academy of Sciences, 142290 Pushchino, Moscow Region, Russia; 5 Key Laboratory of Proteomics and Laboratory of Molecular Cell Biology, Institute of Biochemistry and Cell Biology, Shanghai Institutes for Biological Sciences, Chinese Academy of Sciences, Shanghai, PR China

## Abstract

**Background:**

Protein interactions are essential for most cellular functions. Interactions mediated by domains that appear in a large number of proteins are of particular interest since they are expected to have an impact on diversities of cellular processes such as signal transduction and immune response. Many well represented domains recognize and bind to primary sequences less than 10 amino acids in length called Short Linear Motifs (SLiMs).

**Results:**

In this study, we systematically studied the evolutionary conservation of SLiMs recognized by SH2, SH3 and Ser/Thr Kinase domains in both ordered and disordered protein regions. Disordered protein regions are protein sequences that lack a fixed three-dimensional structure under putatively native conditions. We find that, in all these domains examined, SLiMs are more conserved in disordered regions. This trend is more evident in those protein functional groups that are frequently reported to interact with specific domains.

**Conclusion:**

The correlation between SLiM conservation with disorder prediction demonstrates that functional SLiMs recognized by each domain occur more often in disordered as compared to structured regions of proteins.

## Background

Selective protein-protein interactions are important for many cellular functions and are often mediated by short regions, but such regions are difficult to identify because of their short lengths and degenerate sequences. A significant advance came when peptide-library methods were developed to identify sequences recognized by SH2 domains, which is a globular domain that plays important roles in cellular signal transduction. These peptide-library methods did not depend on prior knowledge of interaction sites *in vivo *[1]. Similar peptide library experiments have been performed to map motifs recognized by other domains [2]. Motifs discovered through polypeptide library screening showed remarkable consonance with reported domain interaction sites [1,2]. Such sites later became the basis for Scansite [3,4], a bioinformatics tool developed to predict target sites recognized by specific protein domains.

Attempts have been made to find such binding regions using purely computational approaches. Eukaryotic linear motifs (ELMs) are identified by their over-representation among protein sequences that bind to a common partner [5]. Short linear motifs (SLiMs) are also identified as specific sequence patterns that are over-represented in proteins that bind to a common partner, but the algorithms used to discover SLiMs employ filters to remove homologous proteins whereas the ELM-discovery algorithms do not. Thus, ELMs and SLiMs are both identified as sequence patterns in multiple proteins that bind to a common target, with the SLiM-containing set likely to be entirely non-homologous but with no such restriction on the ELM-containing set.

Traditionally proteins are believed to function in some form of three-dimensional (3D) structure represented by the "lock and key" or by the "induced fit" theory. More and more examples show that some biological functions of proteins require that the protein structure be more flexible. Disordered protein regions are those sequences in protein that do not have rigid three-dimensional structures. In plots of disorder prediction versus residue number, several sharp dips flanked by regions strongly predicted to be disordered in several different proteins were associated with sites that bind to respective protein partners [6]. This observation was independently made somewhat later [7]. Further analysis on such complexes was carried out [8,9], predictors were developed [10,11], and these binding regions were first named molecular recognition elements [11] then molecular recognition features (MoRFs) [8].

MoRFs differ from ELMs and SLiMs in not depending on a specific sequence motif, but rather upon a pattern in a disorder prediction output. Yet, interestingly, recent analysis suggests that linear motifs (LMs) (thus not differentiating between ELMs and SLiMs) show high overlap with MoRFs [12]. Taken all together, these observations suggest that regions of intrinsic disorder often play a role in protein-protein interactions [13-18]. In addition, there are documented cases where the binding of these disordered regions is coupled to their folding [7,19,20].

SLiMs are known to interact with corresponding functional domains, which might be found in a number of unrelated proteins. These interactions are of particular interest as they might produce a widespread impact on diversities of cellular processes. As this paper is dedicated to the analysis of SLiMs recognized by SH2, SH3 and Ser/Thr Kinase domains, these functional modules are briefly introduced below. Some major functional groups frequently associated with these domains are listed in the Table 1.

**Table 1:** Molecular functional groups frequently reported to interact with Domains.

	Molecular function	binding ratio
SH2	Receptor kinase/phosphatase	0.53
	Y kinase-phosphatase	0.51
	Adapter molecule	0.20
	Cell surface receptor	0.14

SH3	Tyr-kinase/phosphatase	0.32
	Adapter molecule	0.18
	Guanine nucleotide exchange factor	0.12
	Cytoskeletal protein	0.11
	GTPase activating protein	0.11

	Molecular function	phospho ratio

Ser/Thr Kinase	Ser/Thr kinase-phosphatase	0.00442
	Cell cycle control protein	0.00397
	RNA binding protein	0.00334
	Transcription factor	0.00320
	Adapter molecule	0.00296
	Structural protein	0.00259
	Transcription regulatory protein	0.00255

The binding ratio is calculated as the percentage of proteins interacting with proteins containing SH2, SH3 domains. The phosphorylatio ratio is calculated as the percentage of serine residues being phosphorylated.

The Src homology 2 (SH2) domain is a prototypical functional module of ~100 amino acids that contains a central anti-parallel β-sheet surrounded by two α-helices [21]. SH2 domains represent the largest class of known phosphotyrosine (pTyr)-recognition domains [22]. These domains bind specific pTyr-containing motifs, which are typically found in complexes as an extended β-strand that lies at right angles to the SH2 β-sheet [23]. The SLiM-SH2 interactions typically couple activated protein tyrosine kinases (PTKs) to a number of intracellular pathways regulating various aspects of cellular communication [24]. Overall, the SH2 domain is an important functional module found in a great variety of proteins regulating functionally diverse processes. Recently, these SH2-containing proteins were classified into 11 functional categories [23]. The illustrative examples of functions modulated by the SH2-containing proteins include signal regulation, tyrosine phosphorylation, control of phospholipids metabolism, small GTPase regulation, gene expression, chromatin remodeling, ubiquitylation, and cytoskeletal organization. Furthermore, some of the SH2-containing proteins serve as adaptors and scaffolds [23].

Src-homology 3 (SH3) domains generally bind to Pro-rich peptides that form a left-handed polyPro type II helix. SH3 domains are small protein modules of ~60 amino acid residues that typically contain five or six β-strands arranged as two tightly packed anti-parallel β-sheets [25]. The linker regions may contain short helices. Two SH3 variable loops, the RT and n-Src loops, flank a SLiM-binding site that consists of a hydrophobic patch that contains a cluster of conserved aromatic residues [26]. Two classes of SH3 domains have been defined, Class 1 and Class 2, which recognize RKXXPXXP and PXXPXR motifs, respectively [27]. An interesting feature of SH3 domains is the palindromic nature of their ligands; i.e. these domains can bind the SLiMs in either orientation [27]. SH3 domains are found in a great variety of intracellular or membrane-associated proteins, e.g., in a number of proteins with enzymatic activity, in adaptor proteins that lack catalytic sequences and in cytoskeletal proteins, such as fodrin and yeast actin-binding protein ABP-1. SH3 domains mediate assembly of specific protein complexes via binding to proline-rich peptides in their respective binding partner. They are involved in cell-cell communication and signal transduction from the cell surface to the nucleus [28]. Interestingly, SH2 and SH3 domains are frequently found together in the same protein. However, certain proteins contain a single SH2 or SH3 domain, while others contain several copies of either domain [25,27]. Some SH2 domains (e.g., Crk SH2 domain) contain specific SH3 domain-binding sites [29], thus linking together SH2- and SH3-mediated regulatory networks.

Protein phosphorylation is one of the most ubiquitous post-translational modifications of proteins, being the most common mechanism of protein function regulation known to date. In eukaryotes, phosphorylation is carried out by protein kinases, which represent about 2% of the proteins encoded by eukaryotic genomes [30-33]. In human genome, kinases are the third most common protein [33]. Protein kinases are key signalling enzymes, that participate in the regulation of multiple cellular responses and have evolved two properties that are essential for their function: sensitive means of regulation and high specificity for substrates [34]. Ser/Thr kinases transfer the terminal phosphate from ATP to a specific Ser or Thr residue on protein substrates. Some illustrative examples of the most crucial Ser/Thr kinases include mitogen-activated protein kinase (MAPK), glycogen synthase kinase 3 (GSK3), cAMP-dependent protein kinase (PKA), phosphorylase kinase, cyclin-dependent kinase (CDK), protein kinase B (PKB) and phosphoinositide-dependent protein kinase-1 (PDK1) families. Early studies on model Ser/Thr protein kinases revealed that the principal substrate specificity determinants for these kinases were "recognition motifs", located in short segments of the primary sequence around the phosphorylation sites [35,36].

Since protein sequences of functional importance are often highly conserved over evolutionary timescales, it is reasonable to compare the SLiM sequences in both ordered and disordered protein regions by studying their sequence conservation. The supposition is that greater sequence conservation will be observed for functional as compared to non-functional SLiMs. In this study we systematically analysed the conservation of SLiMs recognized by SH2, SH3 and Ser/Thr kinase domains (amino acid residues critically invariant for each domain are shown in Table 2) in ordered and disordered protein regions. Compared to SLiMs in structured regions, SLiMs in disordered regions exhibit greater conservation than their flanking sequences. This greater relative conservation suggests that SLiMs in disordered regions are more likely to be biologically relevant binding sites than those sites within ordered regions.

**Table 2:** Invariant amino acid residues in SLiMs recognized by SH2, SH3 and Ser/Thr Kinase domains.

**Domain**	**SLiM**	**length**
SH2	YXXX	4
SH3 Type 1	XXXPXXP	7
SH3 Type 2	XPXXPXX	7
Ser/Thr Kinase	XXXXS/TXXXX	9

## Methods

### Protein classification and sequence data

Protein sequence data was obtained from SwissProt database downloaded from  in November 2005. Reported protein-protein interactions, protein molecular function classifications, biological processes and sub-cellular localizations were according to the Hprd dataset [37], which is a non-redundent manually curated protein database, downloaded in November 2005 from . Phosphorylated sites were obtained from the Phospho.ELM database [38] kindly provided by Francesca Diella in December 2005.

For our protein functional classification analysis we selected all (7248) human proteins that satisfy following criteria: (i) Each protein had sequence annotated by SwissProt; (ii) Each protein had molecular function annotated by Human protein reference database (Hprd) [37]; (iii) The function of the protein is within 34 protein functional groups in Hprd, all of which are found 50 or more times in Hprd.

### Selection of homologous proteins

Using 7248 human protein sequences selected as described above, we did a BLAST search against 12 other higher eukaryotic species (*Canis familiaris, Bos taurus, Mus musculus, Rattus norvegicus, Gallus gallus, Xenopus tropicalis, Tetraodon nigroviridis, Danio rerio, Strongylocentrotus purpuratus, Drosophila melanogaster, Apis mellifera*, and *Caenorhabditis elegans*) to obtain sequences homologous to the human protein examples. Species were selected according to their unique evolutionary positions (four mammals, four non-mammal vertebrates and four invertebrates) and sequence availability in the RefSeq database [39]. Sequence data for all non-human species were from RefSeq database downloaded from  in June 2006 except for *Tetraodon nigroviridis *which was from the NCBI Entrez non-redundant protein sequence database downloaded from  in June 2006. We applied two cutoff levels to avoid inclusion of insignificant hits: a score cutoff of 50 bits, and an overlap cutoff of 50%, as applied in Inparanoid [40]. If more than one homologous sequence were obtained from a single species, the one with the lowest E-value was selected for this study. However, different from Inparanoid [40] or COG (Cluster of Orthologous Groups) [41], which consider all species as equal entries, because most biochemical data we used including protein interaction data and protein classification data were from human, sequences from all other species were compared to those of human. Therefore, we only considered the best hit from non-human species as homologous to human query protein but not necessarily mutually best matches between human and non-human species or non-human species themselves. Sequence alignments were manually checked and modified when necessary.

### Disorder predictions

Predictions of intrinsic disorder from protein sequence were carried out using a well-characterized disorder predictor VL3 [42,43], which is publicly accessible at our web site . This predictor is trained on the experimentally (X-ray and NMR) confirmed disordered protein regions, while the ordered training set included completely ordered protein regions extracted from the non-redundant set of proteins from PDB Select 25. The accuracy of this predictor, benchmarked on the 42 CASP5 targets, reached 78%. The result is best on all measures, on both no-density segments and B-factors, and is significantly better than the predictors from other groups that participated in CASP5 [44].

### Calculation of the conservation score of SLiM

SLiMs that have amino acid residues critically invariant for each domain (as shown in Table 2) were obtained for evolutionary analysis (Thr-SLiMs were not included in the analysis for Ser/Thr kinases domains since we only have peptide library mapped motifs for Ser-SLiMs). For a particular protein sequence assume sequence identity rate between a reference species (human in this study) and species i is p_(i) _(equal to the number of identical sites divided by the total number of sites aligned), and the SLiM under study is n amino acids in length (in cases where the SLiM is at the terminal of a protein and is only partially available, the available length is considered). If the SLiM is under the same evolutionary selectivity as the full-length protein, then the probability that the SLiM is conserved between the two species is given by:

P_1_(i) = p(i)^n^

The probability that the SLiM is unconserved is given by:

P_2_(i) = 1 - P_1_(i) = 1 - p(i)^n^

Here we define Relative Conservation (C_R_) between human and the i^th ^species as:

a. if the SLiM is conserved:

C_R_(i) = 1/P_1_(i) = 1/p(i)^n^;

b. if the SLiM is unconserved:

C_R_(i) = P_2_(i) = 1 - p(i)^n^;

If C_R_(i) from k different species are [C_R_(1), C_R_(2), C_R_(3),..., C_R_(k)], then C_R _of the SLiM among different species is given by:

$${\text{C}}_{\text{R}}=\sqrt{\prod_{\text{i}=1}^{\text{k}}{\text{C}}_{\text{R}}(\text{i})}$$

A C_R _score greater than 1 indicates the SLiM is C_R _times more conserved than the average level of the protein. A score smaller than 1 indicates 1/C_R _times greater variability between species.

This relative conservation approach is originally developed to study domain recognized motifs within protein sequences in different functional groups (Ren & Chen et al submitted). The method may not be suitable for SLiMs longer than 10 amino acids, since it assumes that most residues in the SLiM could influence the interaction. This may not be the case in longer sequences where only a small subset of the residues is critical to binding. Although not all residues in a SLiM shorter than 10 amino acids are essential for interaction, their relative conservation is usually strong enough to be detected.

Please see Additional file 1 for information on additional materials and methods.

## Results

### Methodology

Traditional methods measure sequence conservation without considering the conservation background of the protein. Here, we took background conservation into consideration by measuring the relative conservation score. Our central hypothesis was that SLiMs should be subject to two kinds of evolutionary selection. The first is background selection, which is imposed upon the entire length of the protein sequence, due to the integral function of the protein. The second is SLiM-specific selection superimposed on the background, due to the special function mediated by the SLiM.

**Figure 1:**
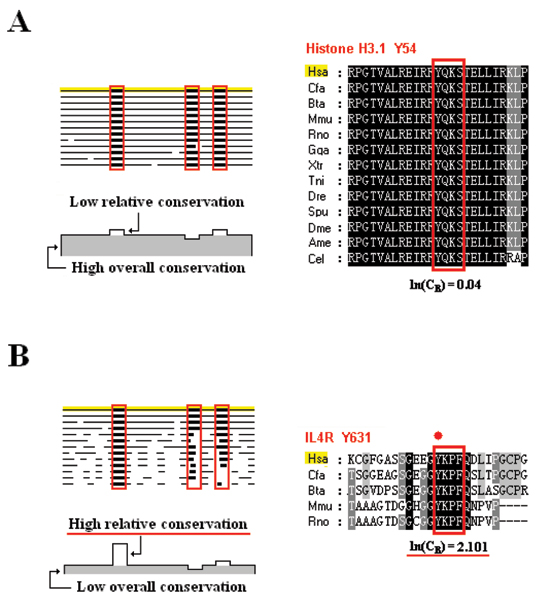
Relative conservation of SLiMs. (A) Low relative conservation of conserved SLiM in overall conserved protein. Schematic illustration (left panel) and alignment (right panel) around Y54 of Histone H3.1. (B) High relative conservation of conserved SLiM in overall less conserved protein. Schematic illustration of relative conservation (left panel) and alignment (right panel) around Y631 of IL4R.

Therefore, a well-conserved SLiM in an overall highly conserved protein does not guarantee independent importance. In this case, the high sequence matching probably results because the SLiM is an integral part of the conserved protein structure. For example, although the putative SH2 binding Tyr-SLiM in Histone H3.1 is conserved among sequences from all selected species, their relative conservation was low because of the highly conserved background (Figure 1A). Conversely, a high relative conservation is an indication that the given SLiM motif may play an important physiological role. As shown in Figure 1B, the Tyr-SLiMs in the C-terminal of IL4R are well conserved while the full-length protein is not so well conserved, and thus these SliMs exhibit a high relative conservation score (Figure 1B). In fact, this tyrosine motif is reported to bind to SH2 domain [45]. Thus, the advantage of the relative conservation method is the capability to discriminate SLiMs conserved under constraints of the integral protein from those conserved to serve as functional motifs. Conserved motifs in conserved proteins might or might not be important; when the SLiM and its protein environment exhibit similar degrees of conservation there is simply no information regarding potential importance. Such SLiMs are reasonably considered to be less likely to function independently compared to those SLiMs that are more conserved than their surrounding sequences.

### Analysis of SH2 domain recognized SLiMs in 11 most studied Receptor Tyrosine Kinases (RTKs)

In this section and the sections that follow, we use "SLiM conservation" to indicate relative conservation unless specified otherwise.

**Figure 2:**
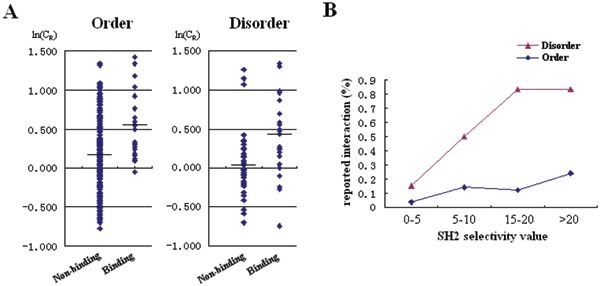
Conservation score and SH2 selectivity values of Tyr-SLiMs in disordered and ordered protein regions in 11 most studied RTKs. (A) SH2 binding Tyr-SLiMs are significantly more conserved than those that do not bind to SH2 domains in both order and disorder groups (both p < 0.001, Mann-Whitney test). (B) Percentage of SLiMs that reported to interact with SH2 domains.

To test our SLiM conservation calculation and its relation with protein disorder, we analyzed the SH2 binding sites reported for 11 highly-studied RTKs (with greater than 30 interaction partners, according to Hprd), including EGFR, IR, KIT, PDGFRB, IGF-IR, VEGFR2, ERBB2, FGFR1, HGFR, RET and TKR-A (for more details, see the Additional file 1). We manually extracted the interactions between these 11 RTKs and 21 SH2 domains from literature. This resulted in a total of 76 interactions involving 56 unique Tyr-SLiMs (see Table S2 for details). Using our SLiM conservation calculation, we found that SLiMs reported to bind to SH2 domains have significantly higher ln(C_R_) scores than those SLiMs that do not bind to SH2 domains in both disordered and ordered sequences (Mann-Whitney test p < 0.001 in both cases, Figure 2A). However, the percentage of SLiMs that bind to SH2 domain differ significantly between disordered and ordered sequences (Figure 2B). We show that with the increase of SH2 selectivity value, the percentage of SLiMs that bind to SH2 domain in disordered protein regions increased to more than 80% under upper medium and high SH2 selectivity values. On the other hand, the percentage of SLiMs that bind to SH2 domain in ordered regions remained below 30% even under high SH2 selectivity value. These results demonstrate that our methods for calculating the conservation score for SLiMs and for predicting domain binding to SLiMs based on motifs from peptide library experiments are effective. Furthermore, our results also show that at least in those 11 most studied RTKs, SLiMs that are within disordered regions are more likely to bind to SH2 domains than those within structured regions.

### Short Linear Motifs recognized by SH2, SH3 and Ser/Thr kinases domains are conserved in disordered regions

**Figure 3:**
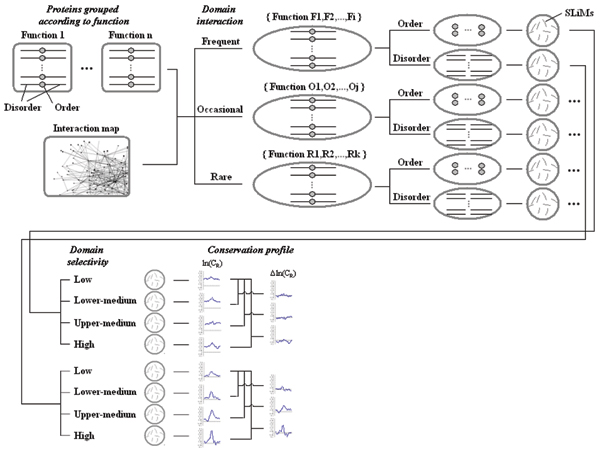
Schematic of the comparion among conservation profiles of SLiMs. For a particular domain that are under study, proteins are first grouped according to their molecular functions then further grouped into three categories according to the involvement of interaction with that domain (frequent, occasional or rare). In each of the categories obtained from the last step, proteins sequences are sorted into ordered and disordered regions. We then get the SLiMs in the protein regions and further grouped them into low, lower medium, upper medium and high domain selectivities. Conservation profiles are calculated for SLiMs in each group. The final output is the difference of ln(C_R_) value between SLiM that with medium, upper medium and high selectivity value and that with low selectivity value.

To investigate the functional importance of domain-recognized SLiMs in ordered and disordered regions of proteins, we performed a systematic analysis on the evolutionary conservation of SLiMs in predicted ordered and disordered protein sequences from different protein functional groups. As shown in Figure 3, for a given domain under study, proteins were first grouped according to their molecular functions then further grouped into three categories according to the involvement of interaction with that domain (frequent, occasional or rare). In each of the categories obtained from the last step, proteins sequences were sorted into ordered and disordered regions according to disorder predictor VL3 (see Methods for details). The SLiMs in both ordered and disordered protein regions were further grouped into low, lower medium, upper medium and high domain selectivity values (See Additional file 1 for details). Conservation profiles were calculated for SLiMs in each group. The final output was the difference of ln(C_R_) values between SLiMs with lower medium, upper medium and high selectivity values as compared to those SLiMs with low selectivity values. The conservation profiles were first averaged within each protein functional group, and then over the different functional groups within frequent, occasional and rare domain binding categories to avoid over-representation of any particular functional groups.

**Figure 4:**
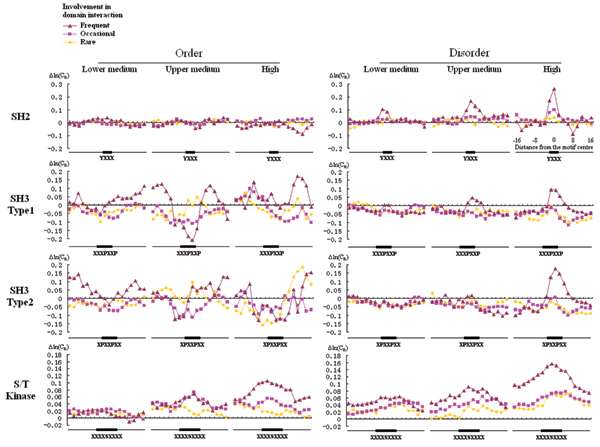
Conservation profiles of Short Linear Motifs (SLiM). Conservation profiles of SLiMs with lower medium, upper medium and high selectivity values for SH2, SH3 and S/T Kinase domains in functional groups that are frequent, occasional or rare interaction partners of each domain.

The frequent, occasional or rare interaction groups for each domain were defined by setting thresholds of the percentage of proteins in the functional group that interact with (or are phosphorylated by) proteins containing that domain according to Hprd (or PhosphoELM) database (see Additional file 1 for details). As expected, in those functional groups that are frequently reported to interact with respective domains, the conservation signal is highest in the motif region that mediates the interaction (Figure 4). Furthermore, conservation signal is highest in frequent binding partners while progressively lowered from occasional to rare binding partners.

Although the conservation of the SLiMs is more manifest in disordered than ordered protein regions in all three domains examined, there are still some differences among the three domains. Tyr-SLiMs recognized by SH2 domains are conserved in disordered but not in ordered protein regions. Ser-SLiMs (since we only had motif with a central Serine residue, only Ser-SLiMs but not Thr-SLiMs were analysed) recognized by Ser/Thr kinases are conserved in both ordered and disordered protein regions but are more conserved in disordered regions. PXXP containing SLiMs recognized by SH3 domains are conserved in disordered but not ordered protein regions. Interestingly, the sequences nearby the PXXP motifs recognized by SH3 have high conservation score. One possible explanation is that the proline residue is strongly disorder-promoting [46,47], and so a structured sequence containing a PXXP motif would be expected to be an unstable element in the rigid structure. In order to compensate for the loss of structural stability brought about by the PXXP motif, the neighbouring residues would become more important for the maintenance of the stability, which may explain their evolutionary conservation.

## Discussions

Protein disorder is believed to play an important role in protein-protein interactions. In this study, we show that the SH2, SH3 and Ser/Thr Kinase domain-recognizable short linear motifs in disordered regions of proteins are more conserved than those in ordered protein regions. This difference is most significant in those molecular functional classes that are frequently reported to interact with their respective domains, but weak in functional groups that are rarely reported to interact with their respective domains.

From an evolutionary perspective, ordered or structural regions are generally more conserved than disordered regions [48]. In this study, calculating the relative conservation of sequences enabled the detection of a conservation signal of a SLiM compared to the conservation background of the protein in which the SLiM resides.

The enrichment of relatively conserved SLiMs in disordered protein regions is highly related to their function. Location of SLiMs in intrinsically disordered regions provides several important functional benefits for interactions with domains. First, SLiMs in disordered regions are more accessible to domains since they are necessarily fully exposed. Second, SLiM domain interaction are usually very weak due to small recognition surface involved. Localization within intrinsically disordered proteins allows the SLiM to adapt to recognition surface and thus improve the stability of the interaction. Third, being located within disordered regions enables overlapping SLiMs to change their conformations to bind to different partners and thus increase signalling complexity. For example, the SH2 domain binds to Tyr-SLiMs previously phosphorylated by Tyr-kinases, so the same region has overlapping motifs, one for the kinase and one for the SH2. The structure of this region changes when it binds to the different partners, and this structural change is facilitated by the flexibility of intrinsic disorder.

Phosphorylation is an important post-translational modification that merits closer attention. Phosphorylation occurs in ~30–50% of the proteins in eukaryotes [49]. Sites of phosphorylation usually occur in disordered regions [50]. Several of SLiMs analyzed in this paper are phosphorylated and we have established that the domain-recognized SLiMs are preferably located in disordered protein regions. Therefore, the results of our analysis support this previous work and *vice versa *– the previous work supports our finding.

Furthermore, several computational methods have been developed for identifying protein phosphorylation sites according to their surrounding peptide sequences. Some of these methods (including NetPhos [51], NetPhosK [52], PredPhospho [53], GPS [54], PPSP [55], ScanSite [9] and Phospho.ELM [38]) depend on datasets of both phosphorylated and non-phosphorylated peptide sequences for training and therefore relying on specific sequence motifs, whereas DisPhos [50] uses disorder, but does not use sequence motifs.

If phosphorylation does indeed occur in disordered regions, then phosphorylation predictors based on the sequence motifs would give a false positive whenever the motif is in a region of structure. That is, if a sequence motif is in a structured region of a protein, the site would be hard to phosphorylate since it does not have the flexibility to fit onto the active site of the kinase (note: binding to the active site requires extended structure and accessible backbone hydrogen bonds, which are hallmarks of disordered proteins [50])

On the other hand, it would be expected that DisPhos would give a false positive when the Ser/Thr or Tyr in a disordered region is not within a kinase recognition motif. These observations suggest that combining a motif-based prediction method with a disorder-based prediction method should give a large increase in phosphorylation prediction accuracy because each method would reduce the false positives from the other method.

This hypothesis was recently confirmed by an elegant study where a new method named PhoScan was elaborated to predict phosphorylation sites for specific protein kinases without using non-phosphorylated training data [56]. The authors have combined both the common (or disorder-based) and the kinase-specific feature sets and added new features that were identified from the training data of known phosphorylation sites. Among these new added features there was the flexibility (disorder) tendency of the local regions surrounding phosphorylation sites evaluated using approach of Iakoucheva *et al*. [50]. PhoScan was shown to achieve a specificity of > 90% and sensitivity ~90% at kinase-family level [50]. This represents a very large improvement compared to the previous methods (about 20%), which likely occurs because the motif-based approach reduces the false positives of the disorder-based approach and vice versa.

Although the SLiM conservation signal is more evident in disordered than ordered protein regions in all the three domains examined, some SLiMs in ordered regions can also interact with domains under physiological condition. For example, serine residues in the structured activation loop of several kinases can be phosphorylated and change the kinase activities. However, these loops undergo large-scale conformational shifts following phosphorylation, and so it is likely that the loops become disordered during the phosphorylation event. This observation suggests that each example in which a motif is apparently in a structured region should be checked for the possibility of transient disorder during binding. Use of transient disorder for signalling presents a number of opportunities for regulation and control [57]. This study has a limited coverage of domains that can interact with SLiMs in the genome. In the future it should be possible to examine other domains-recognized SLiMs using available sequence motifs.

## Conclusion

This study provides evolutionary evidence for the importance of intrinsic disorder in the context of functional protein interactions. Specifically, SLiMs within disordered protein regions are more conserved than equivalent sites within ordered regions. Study of manually extracted SH2 interaction sites in 11 most studied receptor tyrosine kinases provided experimental evidence that Tyr-SLiMs within disordered regions are more likely to be involved in interaction. Although there is currently no direct evidence to show that this is the general rule for SLiMs recognized by domains studied here or other domains in vivo, we hope our current observations will contribute to discussion of the role of intrinsically disordered protein regions.

## Competing interests

The authors declare that they have no competing interests.

## Authors' contributions

SR was involved in design and planning of the experiments has done the computational analysis, designed figures and contributed to the manuscript writing. ZC was involved in planning of experiments, contributed to the manuscript writing and revised the final version. VNU, AKD, and ZO were involved in design and planning of all the experiments, drafted the manuscript and headed the project. All authors have read and approved the final manuscript.

## Supplementary Material

Additional file 1 Click here for file
