# Supplementary material for: Short Linear Motifs recognized by SH2, SH3 and Ser/Thr Kinase domains are conserved in disordered protein regions
Source: BMC Genomics. 2008 Sep 16;9(Suppl 2):S26. doi: 10.1186/1471-2164-9-S2-S26 (PMC2559891; doi:10.1186/1471-2164-9-S2-S26)
Supplement: Additional file 1 [file 1471-2164-9-S2-S26-S1.pdf]

ADDITIONAL MATERIALS AND METHODS

SH2, SH3 and S/T Kinase Motifs used in this study

21 SH2-binding motifs analyzed were Src, Fyn, Lck, Fgr, Abl, Crk, Nck, p85N, p85C, PLCγ1N, PLCγ1C and SHPTP2N [1]; Csk, 3BP2, Fes, GRB2, SHC, Syk and Vav [2]; Shb [3]; and Itk [4]. 8 SH3 motifs are Src, Yes, Abl, Cortactin, p53bp2, PLCγ, Crk and Grb2 [5]; 10 Ser/Thr kinase motifs selected in this study (mainly in basophilic group which is the largest Ser/Thr kinase group) are NIMA, PhK, CamK, [6], PKA, SLK1 [2], AKT [7], PKC [8], SRPK2 [9], MAPKAPK-2 [10] and CLK2 [11].

Calculation of domain and kinase selectivity values

For a putative SLiM, the selectivity value for domains were calculated as the product of enrichment values from peptide library experiments [10, 12]. For example, to calculate the Src SH2 selectivity value of the SLiM YENF, we found the enrichment values for E(Y+1) and N(Y+2) for Src SH2 (Table S1) are 2.5 and 2.4, respectively. No enrichment value for F(Y+3) was found (thus Y+3 does not contribute to the final value) and the selectivity value is the product of the two enrichment values (2.5 x 2.4 = 6.0). The enrichment values for SH3 domain recognized motifs were assigned based on amino acid sequence of peptides expressed by SH3-binding phage clones [5].

Definition of frequent, occasional and rare binding partner (substrate) group

For SH2 and SH3 domains, frequent, occasional and rare binding partner groups are defined by setting thresholds of the percentage of proteins in the functional group that interact with proteins containing that domain according to Hprd data set [13]. The thresholds are >10%, 1-10% and <1% for frequent, occasional and rare binding partners respectively. For S/T Kinases, we set thresholds with the ratio of Serine phosphorylation according to PhosphoELM [14] >2.5‰, 1-2.5‰, <1‰ for frequent, occasional and rare substrate groups respectively. In order to make the results more reliable, we excluded those protein functional groups that have fewer than 300 YXXX motifs for SH2 domains (26 functional groups remained), fewer than 150 PXXP motifs for SH3 domains (31 functional groups remained) and fewer than 2000 Ser-SLiMs for S/T Kinase domains (23 functional groups remained).

Table S1 Enrichment values for the Src SH2 domain

PY+1	pY+2	pY+3
E(2.5)	E(2.6)	I(3.6)
D(1.7)	N(2.4)	M(2.5)
T(1.7)	Y(2.0)	L(2.3)

Table S2 Reported SH2 binding sites in 11 most studied RTKs

Sites	ln(Cr)	Sequence	SH2 domain (reference)
**EGFR**			
727	0.494	FGTVYKGLW	Shc (Schulze WX et. al. 2005)
915	0.763	GSKPYDGIP	Src (Stover DR. et. al. 1995)
944	0.763	TIDVYMIMV	p85 (Stover DR. et. al. 1995)
978	0.564	DPQRYLVIQ	Shp2 (Schulze WX et. al. 2005)
998	0.203	DSNFYRALM	Shc (Schulze WX et. al. 2005), Shp2 (Schulze WX et. al. 2005), Crk (Schulze WX et. al. 2005)
1016	0.004	DADEYLIPQ	PLCg1(Rotein et. al. 1992), Shp2 (Schulze WX et al. 2005)
1092	-0.239	PVPEYINQS	GRB2 (Batzer et. al. 1994, Schulze WX et. al. 2005)
1110	0.243	QNPVYHNQP	GRB2 (Okutani et. al. 1994, Schulze WX et. al. 2005)
1125	-0.742	RDPHYQDPH	GRB2 (Schulze WX et al. 2005)
1138	0.558	GNPEYLNTV	GRB2 (Schulze WX et al. 2005)
1197	-0.049	ENAEYLRVA	Shc (Sakaguchi et. al. 1998, Schulze WX et. al. 2005), PLCg1 (Chattopadhyay et. al. 1999)
**ERBB2**			
735	0.339	FGTVYKGIW	Shc (Schulze WX et. al. 2005)
952	0.548	TIDVYMIMV	p85 (Ram TG et. al. 1996)
1005	0.153	DSTFYRSLL	Shc (Schulze WX et. al. 2005)
1023	0.873	DAEEYLVPQ	Shp2 (Schulze WX et. al. 2005)
1139	0.472	PQPEYVNQP	GRB2 (Ricci A. et. al. 1995, Schulze WX et. al. 2005)
1196	-0.271	ENPEYLTPQ	Shc (Schulze WX et. al. 2005)
1222	0.234	DNLYYWDQD	Shc (Schulze WX et. al. 2005)
1248	0.969	ENPEYLGLD	Shc (Ricci A. et. al. 1995, Schulze WX et. al. 2005)
**FGFR**			
463	0.433	GVSEYELPE	Crk (Larsson, H et. al. 1999)
730	0.099	TNELYMMMR	PLCg1 (Mohammadi M et. al. 1991)
766	0.694	SNQEYLDLS	Shb (Cross MJ et. al. 2002), PLCg1 (Mohammadi M et. al. 1991)
**IGFIR**			
973	0.588	NGVLYASVN	Crk (Koval AP et. al. 1998) , Csk (Arbet-Engels C. et. al. 1999)

980	0.182	VNPEYFSAA	Crk (Koval AP et. al. 1998)
1346	0.583	ERQPYAHMN	Csk (Arbet-Engels C. et. al. 1999), p85 (Seely BL. et. al. 1995), Shp2 (Seely BL. et. al. 1995),
**IR**			
1185	1.045	TRDIYETDY	Shp2 (Kharitonenkov A et. al. 1995)
1361	0.255	EHIPYTHMN	Shp2 (Kharitonenkov A et. al. 1995), Csk (Arbet-Engels C. et. al. 1999), p85 (Van Horn DJ. et. al. 1994)
**KIT**			
568	0.926	NGNNYVYID	Lck (Krystal GW et. al. 1998), Shp2 (Kozlowski M. et. al. 1998)
570	0.926	NNYVYIDPT	Lck (Krystal GW et. al. 1998)
703	0.160	EAALYKNLL	Grb2 (Thommes K et. al. 1999)
721	0.312	STNEYMDMK	p85 (Herbst R et. al. 1995)
900	0.310	PAEMYDIMK	Crk (Lennartsson J et. al. 2003)
936	0.298	TNHIYSNLA	Grb2 (Thommes K et. al. 1999)
**MET**			
1313	0.559	PDPLYEVML	p85 (Maulik G. et. al. 2002)
1349	0.270	IGEHYVHVN	Shc (Pelicci G. et. al. 1995)
1356	0.270	VNATYVNVK	GRB2 (Ponzetto C. et. al. 1996), Shc (Pelicci G. et. al. 1995), Shp2 (Fixman ED. et. al. 1996), PLCg1 (Fixman ED. et. al. 1996)
**PDGFR**			
579	1.340	DGHEYIYVD	Src (Mori S et. al. 1993), Shc (Yokote K et. al. 1994)
581	1.340	HEYIYVDPM	Src (Mori S et. al. 1993)
716	0.243	SAELYSNAL	GRB2 (Amidsson AK et. al. 1994)
740	1.340	SDGGYMDMS	p85 (Panayotou G et. al. 1992), Shc (Yokote K et. al. 1994)
751	1.340	ESVDYVPML	p85 (Panayotou G et. al. 1992), Shc (Yokote K et. al. 1994)
771	0.243	ESSNYMAPY	Shc (Yokote K et. al. 1994)
1009	0.500	SSVLYTAVQ	Shp2 (Lechleider RJ et. al. 1993)
1021	1.340	GDNDYIIPL	PLCg1 (Ronnstrand L et. al. 1992)
**RET**			
981	-0.101	SEEMYRLML	Src (Encinas M et. al. 2004)
1015	1.306	KRRDYLDLA	PLCg1 (Borrello, M et. al. 1996)
1062	0.983	ENKLYGMSD	Shc (Asai N et. al. 1996)
1096	1.185	NDSVYANWM	GRB2 (Alberti, L et. al. 1998)
**TKRA**			
496	0.123	ENPQYFSDA	Shc (Obermeier A et. al. 1993)
680	1.420	YSTDYYRVG	Grb2 (MacDonald JI, et. al. 2000)
681	0.927	STDYYRVGG	Grb2 (MacDonald JI, et. al. 2000)
757	0.098	PPEVYAIMR	p85 (Obermeier A et. al. 1993)
791	0.094	APPVYLDVL	Abl (Yano H et. al. 2000), Grb2 (MacDonald JI, et. al. 2000)

**VEGFR2**			
801	0.646	LKTGYLSIV	PLCg1 (Cunningham SA. et. al. 1997)
1175	0.048	DGKDYIVLP	Shb (Holmqvist K. et. al. 2004), PLCg1 (Cunningham SA. et. al. 1997)
1214	0.266	PKFHYDNTA	Nck (Lamalice L et. al. 2006)

1. Songyang Z, Shoelson SE, Chaudhuri M, Gish G, Pawson T, Haser WG, King F, Roberts T, Ratnofsky S, Lechleider RJ *et al*: **SH2 domains recognize specific phosphopeptide sequences**. *Cell* 1993, **72**(5):767-778.
2. Songyang Z, Shoelson SE, McGlade J, Olivier P, Pawson T, Bustelo XR, Barbacid M, Sabe H, Hanafusa H, Yi T *et al*: **Specific motifs recognized by the SH2 domains of Csk, 3BP2, fps/fes, GRB-2, HCP, SHC, Syk, and Vav**. *Mol Cell Biol* 1994, **14**(4):2777-2785.
3. Karlsson T, Songyang Z, Landgren E, Lavergne C, Di Fiore PP, Anafi M, Pawson T, Cantley LC, Claesson-Welsh L, Welsh M: **Molecular interactions of the Src homology 2 domain protein Shb with phosphotyrosine residues, tyrosine kinase receptors and Src homology 3 domain proteins**. *Oncogene* 1995, **10**(8):1475-1483.
4. Bunnell SC, Diehn M, Yaffe MB, Findell PR, Cantley LC, Berg LJ: **Biochemical interactions integrating Itk with the T cell receptor-initiated signaling cascade**. *J Biol Chem* 2000, **275**(3):2219-2230.
5. Sparks AB, Rider JE, Hoffman NG, Fowlkes DM, Quillam LA, Kay BK: **Distinct ligand preferences of Src homology 3 domains from Src, Yes, Abl, Cortactin, p53bp2, PLCgamma, Crk, and Grb2**. *Proc Natl Acad Sci U S A* 1996, **93**(4):1540-1544.
6. Songyang Z, Lu KP, Kwon YT, Tsai LH, Filhol O, Cochet C, Brickey DA, Soderling TR, Bartleson C, Graves DJ *et al*: **A structural basis for substrate specificities of protein Ser/Thr kinases: primary sequence preference of casein kinases I and II, NIMA, phosphorylase kinase, calmodulin-dependent kinase II, CDK5, and Erk1**. *Mol Cell Biol* 1996, **16**(11):6486-6493.
7. Yaffe MB, Leparc GG, Lai J, Obata T, Volinia S, Cantley LC: **A motif-based profile scanning approach for genome-wide prediction of signaling pathways**. *Nat Biotechnol* 2001, **19**(4):348-353.
8. Nishikawa K, Sawasdikosol S, Fruman DA, Lai J, Songyang Z, Burakoff SJ, Yaffe MB, Cantley LC: **A peptide library approach identifies a specific inhibitor for the ZAP-70 protein tyrosine kinase**. *Mol Cell* 2000, **6**(4):969-974.
9. Wang HY, Lin W, Dyck JA, Yeakley JM, Songyang Z, Cantley LC, Fu XD: **SRPK2: a differentially expressed SR protein-specific kinase involved in mediating the interaction and localization of pre-mRNA splicing factors in mammalian cells**. *J Cell Biol* 1998, **140**(4):737-750.
10. Manke IA, Nguyen A, Lim D, Stewart MQ, Elia AE, Yaffe MB: **MAPKAP kinase-2 is a cell cycle checkpoint kinase that regulates the G2/M transition and S phase progression in response to UV irradiation**. *Mol Cell* 2005, **17**(1):37-48.
11. Nikolakaki E, Du C, Lai J, Giannakouros T, Cantley L, Rabinow L: **Phosphorylation by LAMMER protein kinases: determination of a consensus site, identification of in vitro

substrates, and implications for substrate preferences. *Biochemistry* 2002, **41**(6):2055-2066.

12. Songyang Z, Cantley LC: **The use of peptide library for the determination of kinase peptide substrates**. *Methods Mol Biol* 1998, **87**:87-98.
13. Peri S, Navarro JD, Kristiansen TZ, Amanchy R, Surendranath V, Muthusamy B, Gandhi TK, Chandrika KN, Deshpande N, Suresh S *et al*: **Human protein reference database as a discovery resource for proteomics**. *Nucleic Acids Res* 2004, **32**(Database issue):D497-501.
14. Diella F, Cameron S, Gemund C, Linding R, Via A, Kuster B, Sicheritz-Ponten T, Blom N, Gibson TJ: **Phospho.ELM: a database of experimentally verified phosphorylation sites in eukaryotic proteins**. *BMC Bioinformatics* 2004, **5**:79.
